# The role of BRAF testing of Rathke’s cleft cysts to identify missed papillary craniopharyngioma

**DOI:** 10.1007/s11102-025-01501-8

**Published:** 2025-02-03

**Authors:** Nicholas G. Candy, E. Mignone, E. Quick, B. Koszyca, A. Brown, I. M. Chapman, D. J. Torpy, N. Vrodos, S. Santoreneos, S. M. C. De Sousa

**Affiliations:** 1 Department of Surgery – Otolaryngology, Head and Neck Surgery, The University of Adelaide, Basil Hetzel Institute for Translational Research, Woodville South, Adelaide, Australia; 2 Endocrine and Metabolic Unit, Royal Adelaide Hospital, Adelaide, Australia; 3 SA Pathology, Flinders Medical Centre, Adelaide, Australia; 4 SA Pathology, Royal Adelaide Hospital, Adelaide, Australia; 5 Department of Genetics and Molecular Pathology, SA Pathology, Adelaide, SA Australia; 6 Centre for Cancer Biology, SA Pathology, University of South Australia, Adelaide, SA Australia; 7 School of Medicine, University of Adelaide, Adelaide, SA Australia; 8 Department of Neurosurgery, Flinders Medical Centre, Adelaide, Australia; 9 Department of Neurosurgery, Royal Adelaide Hospital, Adelaide, Australia; 10 Adult Genetics Unit, Royal Adelaide Hospital, Adelaide, Australia

**Keywords:** Rathke’s cleft cyst, Papillary craniopharyngioma, BRAF, Endoscopic endonasal surgery, Craniopharyngioma

## Abstract

**Aim:**

The differential diagnosis of cystic sellar/suprasellar lesions includes craniopharyngioma (CP) and Rathke’s cleft cyst (RCC). Histological differentiation between cystic papillary craniopharyngioma (pCP) and RCC using light microscopy alone is challenging. A major point of difference is that virtually all pCPs are clonal for the *BRAF* V600E variant, whereas RCCs are not. Noting that BRAF testing of RCCs is not current standard practice, we hypothesised that routinely performing BRAF studies in RCCs might uncover otherwise missed pCPs.

**Method:**

We performed a retrospective cohort study of all RCCs operated on at Flinders Medical Centre, the Memorial and Royal Adelaide Hospitals, between 2001 and 2023. In cases with sufficient tissue, we performed BRAF V600E immunohistochemistry (IHC) and *BRAF* next generation sequencing (NGS) of extracted tumour DNA.

**Results:**

Of eleven patients with suitable operative specimens, one patient with an initial diagnosis of RCC was revised to pCP following BRAF testing with equivocal positivity on BRAF IHC and clear identification of the V600E variant on NGS. The patient’s subsequent clinical course was aggressive and more compatible with pCP than RCC.

**Conclusion:**

This study highlights the potential value of BRAF testing in RCCs to identify missed pCP, which is an especially timely finding given the advent of primary medical therapy with BRAF inhibition for pCP. In the absence of guidelines advising on the use of BRAF studies in sellar lesions, we suggest consideration of BRAF testing of all RCCs, particularly if there is squamous metaplasia or disease recurrence.

## Background

The differential diagnosis of cystic sellar/suprasellar lesions include Rathke’s cleft cyst (RCC) and craniopharyngioma (CP) [1]. CPs are divided into adamantinomatous (aCP) and papillary CP (pCP), with the former being more prevalent [2]. The molecular basis of CPs has been elucidated with over 95% of pCP harbouring the *BRAF* c.1799T > A, p.(Val600Glu) variant commonly referred to as ‘V600E’ [3, 4], and this variant has recently been targeted with BRAF-MEK inhibitors with profound tumour shrinkage [5].

RCCs are embryological remnants of the Rathke’s pouch which form when there is a failure of involution of the craniopharyngeal duct, leaving a cyst between the pars distalis and the pars nervosa [6]. The cyst wall is lined by simple cuboidal or columnar epithelium with and without cilia, with pseudostratified columnar respiratory epithelium being present in half of cases [7].

Overlapping clinical, radiological and pathological features can make differentiation between pCP and RCC challenging. However, differentiating between the two pathologies is essential given their divergent clinical trajectories with the need for more intensive management and consideration of BRAF inhibition in pCP. Radiological distinction between pCP and RCC is limited as both lesions can appear as simple cystic lesions in the sellar/suprasellar space and pCPs lack the classical calcifications of aCP [8]. Histological distinction between these two entities using light microscopy alone is also challenging [9].

Since the V600E *BRAF* variant is present in almost all cases of pCP, this genetic finding should allow differentiation between RCC with squamous metaplasia and pCP. However, BRAF studies of RCCs are not part of routine practice and the decision to examine BRAF depends on the treating clinician and/or pathologist [5].

The aim of this study was to examine the value of routine BRAF testing in lesions otherwise diagnosed as RCCs. The study was prompted by the finding of the *BRAF* V600E variant in a patient initially thought to have RCC, with his diagnosis subsequently revised to pCP. We then performed a retrospective study applying BRAF testing in a cohort of individuals with resected RCC.

## Methods

The case notes for all patients undergoing endoscopic endonasal surgery performed at Flinders Medical Centre, the Memorial Hospital and Royal Adelaide Hospital between 2001 and 2023 by two senior surgeons were reviewed. Using the histopathology results, cases were screened and patients with a diagnosis of RCC were identified. Clinical and histological data were collected by two authors (NGC and SMCD). Written consent for publication was obtained from the next of kin of the index case. Publication approval for the manuscript was obtained from the Central Adelaide Local Health Network Human Research Ethics Committee. Consent from other participants as well as ethical and governance approval were waived by the HREC in view of the retrospective nature of the audit and only summary data being published.

In patients with RCCs with sufficient tissue, we performed BRAF V600E immunohistochemistry (IHC) and *BRAF* next generation sequencing (NGS) of extracted tumour DNA. IHC to detect the BRAF V600E variant was undertaken using a VE1 antibody consistent with previously described methods [10]. NGS was performed with a targeted gene panel used in tumour testing across different neoplasms.

## Results

### Index case

A 63-year-old man presented to the emergency department with dizziness and an incidental finding on CT imaging of a large cystic sellar and suprasellar mass (L 25 x H 30 mm) with compression of the optic chiasm. MRI confirmed a simple cyst of the sellar and suprasellar region, favouring a Rathke’s cleft cyst (RCC). Biochemical evaluation revealed hypopituitarism and was commenced on hormonal replacement with hydrocortisone and levothyroxine. Humphrey visual field perimetry showed bitemporal hemianopia. He underwent elective endoscopic transsphenoidal resection of his presumed RCC.

**Fig. 1:**
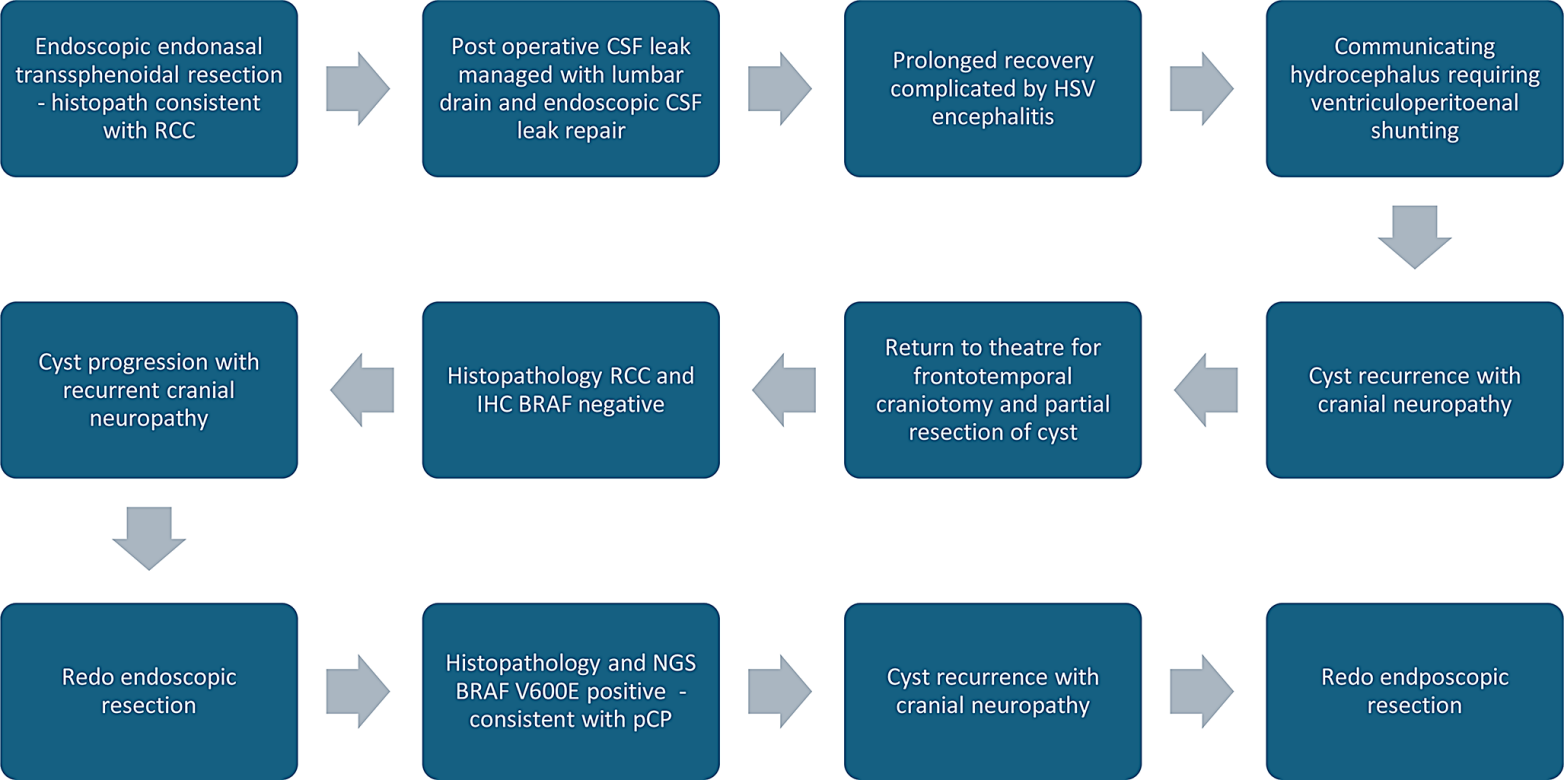
Flow chart demonstrating the significant events during the patient’s management

The patient underwent a complex clinical course, summarised in (Fig. 1). During a revision surgery the histopathology was re-examined for BRAF variants, confirming a V600E mutation. This subsequently revised the patient’s initial diagnosis of RCC to pCP.

### Retrospective cohort study

An additional ten patients with RCC were identified with suitable operative specimens for BRAF testing (total *n* = 11). BRAF IHC was performed in all 11 cases. BRAF NGS was successfully undertaken in 8/11 cases, with insufficient DNA extracted in the remaining three cases. The results of these patients in addition to the index case are displayed in Table 1.

**Table 1:** Case summary of patients who had RCCs resected endoscopically and sufficient tissue to perform BRAF testing. Case 1 is the index case reported herein. BRAF V600E IHC results are displayed for lesional (epithelial) tissue as well as adjacent anterior pituitary gland. The presence of squamous metaplasia in lesional tissue is indicated

Patient number	BRAF IHC tumour/cyst	BRAF IHC anterior pituitary tissue	Squamous metaplasia	BRAF V600E status by NGS	Estimated tumour cell content	Recurrence requiring revision surgery
1	Equivocal	Positive	Positive	BRAF 1799T > A p. (Val600Glu)	50%	Yes
2	Negative	Positive	Negative	Negative	20%	No
3	Negative	Positive	Positive	Negative	10%	No
4	Negative	Positive	Negative	Insufficient	15%	No
5	Negative	Positive	Positive	Negative	10%	Yes
6	Negative	No gland	Negative	Insufficient	30%	No
7	Negative	Positive	Negative	Negative	50%	No
8	Negative	Positive	Negative	Negative	1%	No
9	Positive	Positive	Negative	Negative	10%	No
10	Negative	Positive	Negative	Negative	10%	No
11	Negative	Positive	negative	Insufficient	30%	No

### IHC and NGS results in lesional tissue

All patients had sufficient tissue for BRAF IHC analysis. One of the eleven patients exhibited positive IHC labelling in lesional tissue, and the index case had equivocal IHC labelling. BRAF V600E IHC was negative in the lesional tissue of all other cases.

Eight of the eleven patients had sufficient tissue to proceed with NGS; the remaining three patients had insufficient tissue or insufficient extractable DNA. In the index case with an equivocal IHC result in lesional tissue, NGS demonstrated the *BRAF* c.1799T > A, p. (Val600Glu) variant, thereby upgrading the diagnosis from RCC to pCP. In the case with a positive IHC result in lesional tissue, no *BRAF* variant was identified and thus the diagnosis of RCC was upheld.

The diagnosis of RCC was upheld in all other cases on account of negative BRAF studies. Hence, overall, 1/11 (9%) of RCC cases were revised to pCP through BRAF studies.

### Squamous metaplasia

Squamous metaplasia was noted in three patients, including the index case who was ultimately diagnosed with pCP. The remaining two patients had negative results for the BRAF V600E variant by both IHC and NGS testing. One of these patients underwent revision surgical resection for a recurrence and the other case demonstrates no recurrence at last follow-up 7 years postoperatively.

## Discussion

The index case presented here illustrates the potential value of routine BRAF studies in the evaluation of suspected RCC. Potential alterations in management following a revised diagnosis to pCP include more frequent follow-up, consideration of revision surgery in the appropriately selected patient, and consideration of BRAF/MEK inhibitor therapy which is currently transforming the treatment paradigm of pCP [5]. However, missed pCP in the RCC setting appears to be an uncommon phenomenon as this occurred in only 1/11 (9%) of the overall retrospective cohort.

**Fig. 2:**
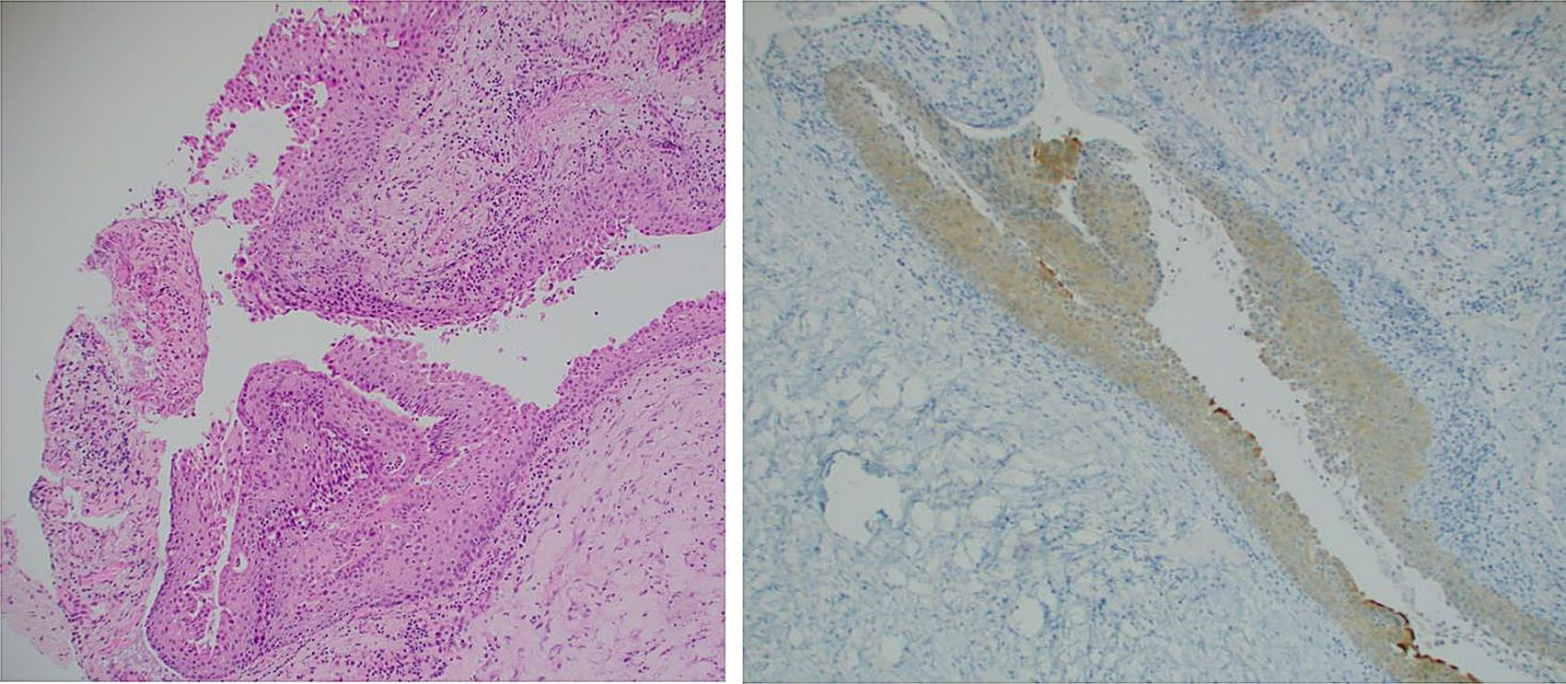
Left image is an H&E stain demonstrating portions of squamous lined cys, with underlying fibrous tissue and a minor acute inflammatory infiltrate. Right image is a BRAF V600E stain demonstrating pale staining of the squamous epithelium but focal darker staining of the normal cilia. Both images at 10x magnification

The index case initially demonstrated a cyst lined by cuboidal and columnar epithelium with apical mucin, without cytological atypia or squamous metaplasia. As such, BRAF IHC was not undertaken. At the first revision surgery repeat specimens demonstrated columnar epithelium with apical mucin again without squamous metaplasia but the presence of a neutrophilic infiltrate. BRAF IHC was negative on these specimens. The second revision surgery demonstrated squamous metaplasia that had weak staining to BRAF IHC and NGS detected a BRAF V600E mutation, confirming the diagnosis of pCP (Fig. 2).

It is possible that the patient’s outcome may have been different had a diagnosis of pCP been made earlier in their treatment course. Therefore, it would be reasonable to consider routine BRAF IHC for non PitNET sellar and suprasellar lesions with molecular BRAF testing considered individually.

Schweizer et al. examined 33 RCC specimens and 18 pCP specimens and found that 30/33 RCC specimens were negative by BRAF IHC. The three cases with RCC and positive BRAF immunochemistry had unusual clinical courses, with two being re-diagnosed as pCP and one having a past diagnosis of a pCP.

These cases demonstrate the role molecular testing can play in the work-up of non-PitNET lesions of the sella. IHC has a fast turnaround time but interpretation within the CNS and pituitary can be difficult with susceptibility to interobserver variation. NGS is a more specific test, but accessibility has traditionally been an issue. Impediments to NGS testing include increased turnaround time (10–21 days vs. 1–2 days for IHC), cost (1,000–4,000 USD vs. 40–140 USD) and tissue requirements (> 8 formalin fixed paraffin embedded (FFPE) sections vs. 1 FFPE section, and 50ng – 2microg DNA/RNA vs. 50 tumour cells) [11]. These limitations are becoming less significant with more efficient NGS and bioinformatic processes that allow turnaround in a similar time frame to IHC and equivalent costs at certain facilities.

In the present study, both IHC and NGS were available, with complementary advantages. IHC was able to be performed in all cases, whereas there was inadequate tissue for NGS in 3/11 cases. On the other hand, NGS offered definitive results in all cases with adequate tissue with no evidence to suggest a false-positive result (as suspected in the positive IHC result in case 9 which lacked other pCP features such as squamous metaplasia and recurrence) or a sampling error result (as suspected in the initial negative IHC result in the index case).

## Conclusion

These results demonstrate the potential value of BRAF testing in distinguishing RCC from pCP. BRAF IHC and *BRAF* NGS are complementary studies with different tissue requirements and assay considerations. We recommend that BRAF studies be considered in all non-PitNET sellar and suprasellar lesions according to institutional resources, but especially in those with squamous metaplasia and/or lesional recurrence and those where BRAF inhibitor therapy or further surgery would be considered if the lesion was proven to be a pCP.

## Data Availability

No datasets were generated or analysed during the current study.

## References

[1] Marucci G, de Biase D, Zoli M, Faustini-Fustini M, Bacci A, Pasquini E et al (2015) Targeted BRAF and CTNNB1 next-generation sequencing allows proper classification of nonadenomatous lesions of the sellar region in samples with limiting amounts of lesional cells. Pituitary 18(6):905–911 26156055 10.1007/s11102-015-0669-y

[2] Diaz MJ, Kwak SH, Root KT, Fadil A, Nguyen A, Ladehoff L et al (2022) Current approaches to Craniopharyngioma Management. Front Biosci (Landmark Ed) 27(12):328 36624954 10.31083/j.fbl2712328

[3] Goschzik T, Gessi M, Dreschmann V, Gebhardt U, Wang L, Yamaguchi S et al (2017) Genomic alterations of Adamantinomatous and Papillary Craniopharyngioma. J Neuropathol Exp Neurol 76(2):126–134 28069929 10.1093/jnen/nlw116

[4] Brastianos PK, Taylor-Weiner A, Manley PE, Jones RT, Dias-Santagata D, Thorner AR et al (2014) Exome sequencing identifies BRAF mutations in papillary craniopharyngiomas. Nat Genet 46(2):161–165 24413733 10.1038/ng.2868 PMC3982316

[5] Brastianos PK, Twohy E, Geyer S, Gerstner ER, Kaufmann TJ, Tabrizi S et al (2023) BRAF-MEK inhibition in newly diagnosed Papillary Craniopharyngiomas. N Engl J Med 389(2):118–126 37437144 10.1056/NEJMoa2213329 PMC10464854

[6] Trifanescu R, Ansorge O, Wass JA, Grossman AB, Karavitaki N (2012) Rathke’s cleft cysts. Clin Endocrinol (Oxf) 76(2):151–160 21951110 10.1111/j.1365-2265.2011.04235.x

[7] Shin JL, Asa SL, Woodhouse LJ, Smyth HS, Ezzat S (1999) Cystic lesions of the pituitary: clinicopathological features distinguishing craniopharyngioma, Rathke’s cleft cyst, and arachnoid cyst. J Clin Endocrinol Metab 84(11):3972–3982 10566636 10.1210/jcem.84.11.6114

[8] Schlaffer SM, Buchfelder M, Stoehr R, Buslei R, Holsken A (2018) Rathke’s Cleft Cyst as Origin of a Pediatric Papillary Craniopharyngioma. Front Genet 9:49 29520296 10.3389/fgene.2018.00049 PMC5826961

[9] Schweizer L, Capper D, Holsken A, Fahlbusch R, Flitsch J, Buchfelder M et al (2015) BRAF V600E analysis for the differentiation of papillary craniopharyngiomas and Rathke’s cleft cysts. Neuropathol Appl Neurobiol 41(6):733–742 25442675 10.1111/nan.12201

[10] Liu H, Li Z, Wang Y, Feng Q, Si L, Cui C et al (2014) Immunohistochemical detection of the BRAF V600E mutation in melanoma patients with monoclonal antibody VE1. Pathol Int 64(12):601–606 25359093 10.1111/pin.12215

[11] Tsao MS, Yatabe Y (2019) Old soldiers Never die: is there still a role for immunohistochemistry in the era of next-generation sequencing panel testing? J Thorac Oncol 14(12):2035–2038 31757371 10.1016/j.jtho.2019.09.007

